# Zinc Prevents DNA Damage in Normal Cells but Shows Genotoxic and Cytotoxic Effects in Acute Myeloid Leukemia Cells

**DOI:** 10.3390/ijms23052567

**Published:** 2022-02-25

**Authors:** Maria Inês Costa, Beatriz Santos Lapa, Joana Jorge, Raquel Alves, Isabel Marques Carreira, Ana Bela Sarmento-Ribeiro, Ana Cristina Gonçalves

**Affiliations:** 1 Laboratory of Oncobiology and Hematology (LOH) and University Clinic of Hematology, Faculty of Medicine (FMUC), University of Coimbra, 3000-548 Coimbra, Portugal; uc2018265624@student.uc.pt (M.I.C.); uc2013143376@student.uc.pt (B.S.L.); jjorge@fmed.uc.pt (J.J.); raquel.alves@fmed.uc.pt (R.A.); absarmento@fmed.uc.pt (A.B.S.-R.); 2 Coimbra Institute for Clinical and Biomedical Research (iCBR), Group of Environmental Genetics of Oncobiology (CIMAGO), Faculty of Medicine (FMUC), University of Coimbra, 3000-548 Coimbra, Portugal; icarreira@fmed.uc.pt; 3 Center for Innovative Biomedicine and Biotechnology (CIBB), 3004-504 Coimbra, Portugal; 4 Clinical Academic Center of Coimbra (CACC), 3000-061 Coimbra, Portugal; 5 Cytogenetics and Genomics Laboratory, Faculty of Medicine, University of Coimbra, 3004-531 Coimbra, Portugal; 6 Hematology Service, Centro Hospitalar e Universitário de Coimbra (CHUC), 3000-061 Coimbra, Portugal

**Keywords:** zinc, DNA damage, DNA repair, genomic instability, acute myeloid leukemia

## Abstract

Genomic instability is prevented by the DNA damage response (DDR). Micronutrients, like zinc (Zn), are cofactors of DDR proteins, and micronutrient deficiencies have been related to increased cancer risk. Acute myeloid leukemia (AML) patients commonly present Zn deficiency. Moreover, reports point to DDR defects in AML. We studied the effects of Zn in DDR modulation in AML. Cell lines of AML (HEL) and normal human lymphocytes (IMC) were cultured in standard culture, Zn depletion, and supplementation (40 μM ZnSO_4_) conditions and exposed to hydrogen peroxide (H_2_O_2_) or ultraviolet (UV) radiation. Chromosomal damage, cell death, and nuclear division indexes (NDI) were assessed through cytokinesis-block micronucleus assay. The phosphorylated histone H2AX (yH2AX) expression was monitored at 0 h, 1 h, and 24 h after exposure. Expression of DDR genes was evaluated by quantitative real time polymerase chain reaction (qPCR). Zn supplementation increased the genotoxicity of H_2_O_2_ and UV radiation in AML cells, induced cytotoxic and antiproliferative effects, and led to persistent yH2AX activation. In contrast, in normal lymphocytes, supplementation decreased damage rates, while Zn depletion favored damage accumulation and impaired repair kinetics. Gene expression was not affected by Zn depletion or supplementation. Zn presented a dual role in the modulation of genome damage, preventing damage accumulation in normal cells and increasing genotoxicity and cytotoxicity in AML cells.

## 1. Introduction

Genome integrity is assured by several signaling molecules, sensors, mediators, transducers, and effector proteins that cooperate to eliminate or minimize DNA damage. [1,2]. These complex protein networks constitute the DNA damage response (DDR). Many micronutrients are substrates and cofactors of key DDR reactions, meaning that their bioavailability is critical for damage recognition and repair [2,3,4,5]. Among several micronutrients, zinc (Zn) is particularly important for playing pivotal roles in the cell. Zn ions associated with proteins influence processes such as antioxidant defenses, DNA replication, transcription, translation, DNA repair, chromatin structure, proliferation, maturation, immune responses, and cell death [6,7,8,9]. Zn is mostly known to support genome stability through its potent antioxidant properties [5,8,9]. However, its effects seem to be multidimensional and include the regulation of many DDR components [5,8,9]. Notably, several DNA repair mechanisms require Zn. Damage sensors and repair proteins involved in base excision repair (BER), nucleotide excision repair (NER), single-strand breaks (SSB), and double-strand breaks (DSB) repair, as well as factors implicated in cell cycle and apoptosis, depend on Zn for regulatory, structural, and catalytic purposes [5,9].

The role of Zn in disease prevention is well defined. However, its functions in carcinogenesis are far less explored. In the context of the hematopoietic system, an appropriate DDR is particularly relevant due to the extended life span of hematopoietic stem cells (HSC) [10]. The DDR avoids the accumulation of DNA damage, which could alter the capacity of self-renewal and the regenerative potential of these stem cells, leading to functional decline and defective hematopoiesis [10]. Recently, DDR alterations have been reported in acute myeloid leukemia (AML) and are related to leukemogenesis [11]. Moreover, a common observation in leukemia patients is a marked decrease in serum Zn levels [12,13]. Despite the frequency of this finding, its biological significance in leukemogenesis and its relationship with the DDR in AML cells is not understood. In this context, we aimed to understand the effects of Zn in the modulation of DDR in AML and normal lymphocytes. For a better understanding of the disease and to improve therapeutic approaches, more attention should be paid to the implications of Zn and DDR alterations in AML.

## 2. Results

### 2.1. Zn Depletion Increased Chromosomal Damage and yH2AX Expression in Normal Lymphocytes and AML Cells without Exposure to Genotoxic Agents

**Figure 1:**
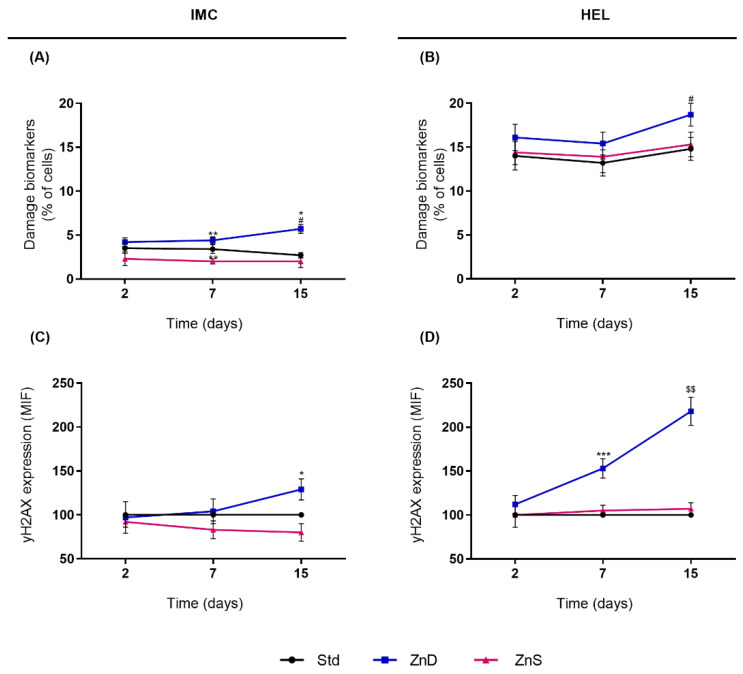
Endogenous genome damage of normal lymphocytes and AML cells in Zn depletion and supplementation. Basal chromosomal damage in IMC (**A**) and HEL cells (**B**) expressed as means ± SEM of the percentage of cells displaying chromosomal damage biomarkers (MNi, NBUDs, and NPBs) from 6 independent experiments. Basal expression of yH2AX in IMC (**C**) and HEL cells (**D**) expressed as means ± SEM of the mean fluorescence intensity (MIF) from 3 independent experiments. MFI of ZnD and ZnS cells were normalized to Std. Note: * refers to comparison to Std, $ to 2nd day, and # to 7th day, with * and # *p* < 0.05, ** and $$ *p* < 0.01, and *** *p* < 0.001. Std, standard; ZnD, Zn-depleted; ZnS, Zn-supplemented.

We started by characterizing damage and cellular responses to Zn depletion and supplementation without exposure to genotoxic agents. Normal lymphocytes (IMC cells) cultured in Zn depletion presented higher scores of damage biomarkers than cells from Std Zn conditions (1.3-fold increase at the 7th day, *p* = 0.010; 2.1-fold increase at the 15th day, *p* = 0.049; Figure 1A). Moreover, the extent of Zn deprivation increased chromosomal damage (1.3-fold increase from the 7th to the 15th day, *p* = 0.023). Contrastingly, supplemented lymphocytes presented 1.7-fold lower scores of chromosomal damage biomarkers on the 7th day than cells from the Std condition (*p* = 0.034). In the AML cell line, HEL, no significant differences in damage rates were noticed between ZnS and Std conditions (Figure 1B). However, when the Zn depletion persisted over time, an increase in chromosomal damage of about 1.2-fold was observed from the 7th to the 15th day of Zn depletion (*p* = 0.047). Expression of yH2AX, a biomarker of DSB, was also assessed (Figure 1C,D). Zn depletion increased yH2AX levels, both in normal lymphocytes and AML cells. In IMC cells cultured for 15 days in Zn depletion, the expression of yH2AX increased 1.3-fold (*p* = 0.029). Similarly, in HEL cells, the yH2AX expression increased 2.7-fold (*p* = 0.006) from the 2nd to the 15th day of Zn depletion (2.7-fold). Supplementation of IMC or HEL cells did not significantly influence yH2AX expression. Nevertheless, a slight decrease in yH2AX expression was observed in IMC cells supplemented with Zn.

### 2.2. Zn Supplementation Differentially Modulated Cell Death in Normal Lymphocytes and AML Cells without Exposure to Genotoxic Agents

**Figure 2:**
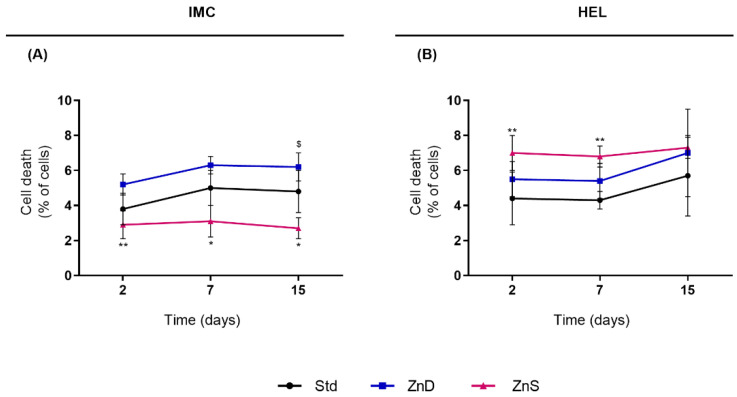
Basal cell death of normal lymphocytes and AML cells in Zn depletion and supplementation. Cell death in IMC (**A**) and HEL cells (**B**), expressed as means ± SEM of the percentages of cells displaying morphological features of apoptosis and necrosis from 6 independent experiments. Note: * refers to comparison to Std and $ to 2nd day, with * and $ *p* < 0.05 and ** *p* < 0.01. Std, standard; ZnD, Zn-depleted; ZnS, Zn-supplemented.

The percentage of normal lymphocytes (IMC) displaying morphological features of cell death increased through time on cells subjected to Zn depletion (1.2-fold, *p* = 0.042), whereas supplementation decreased the cell death count (Figure 2A). Moreover, there were slight, despite insignificant, decreases in cell proliferation in ZnD IMC cells (Table 1). In HEL cells not exposed to genotoxic agents (Figure 2B), Zn deprivation did not significantly affect cell death, whereas supplementation increased cell death scores by about 1.6-fold after 2 and 7 days of Zn supplementation comparatively to Std condition (*p* = 0.007 and *p* = 0.002, respectively).

The proliferation rates of HEL cells decreased both in the absence and presence of Zn (Table 1).

### 2.3. Zn Supplementation Protected Normal Lymphocytes from Chromosomal Damage Following Genotoxic Exposure but Potentiated Genotoxicity in AML Cells

**Figure 3:**
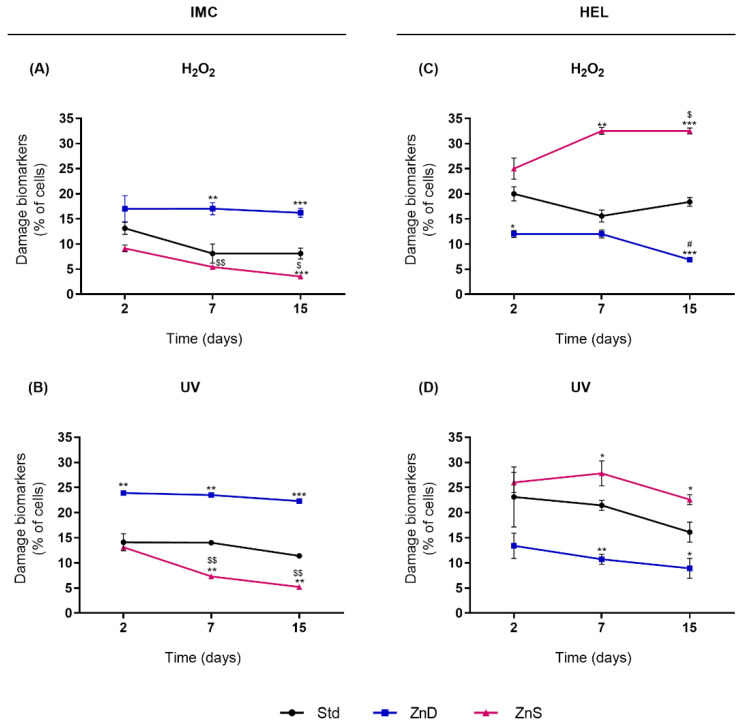
The effects of Zn depletion and supplementation in the modulation of chromosomal damage following genotoxic exposure in IMC (**A**,**B**) and HEL cells (**C**,**D**). Results are expressed as means ± SEM of the percentage of cells displaying chromosomal damage biomarkers (MNi, NBUDs, and NPBs) from 6 independent experiments. Note: * refers to comparison to Std, $ to 2nd day, and # to 7th day, with *, $ and # *p* < 0.05, ** and $$ *p* < 0.01, and *** *p* < 0.001. Std, standard; ZnD, Zn-depleted; ZnS, Zn-supplemented.

Subsequently, we studied the effects of Zn in the modulation of the DDR following genotoxic exposure. Different profiles of chromosomal damage were observed depending on the Zn content and cell line (Figure 3). In normal lymphocytes (IMC), the percentage of cells presenting chromosomal damage biomarkers increased significantly following H_2_O_2_ and UV exposure in Zn depletion, whereas cells cultured in Zn supplementation displayed significantly lower damage rates (Figure 3A,B). Furthermore, in these cells, damage biomarkers after genotoxic exposure decreased in a time of supplementation-dependent manner. Specifically, scores of damage biomarkers were 2.6-fold lower (*p* = 0.034) in H_2_O_2_-exposed lymphocytes after 15 days of Zn supplementation compared to cells supplemented for 2 days. The same was observed for UV-irradiated IMC cells, where a 2.5-fold decrease was detected (*p* = 0.047). A contrasting profile was observed in HEL cells, in which damage rates following genotoxic exposure were higher in supplemented cells and lower in the ZnD cells (Figure 3C,D). Particularly, after 15 days of Zn supplementation, exposure to H_2_O_2_ and UV radiation increased chromosomal damage by 1.7-fold (*p* < 0.001) and 1.3-fold (*p* = 0.021), respectively, compared to cells in Std condition. Moreover, chromosomal damage after exposure of HEL cells to H_2_O_2_ increased with time of Zn supplementation (1.3-fold increase from the 2nd to the 15th day, *p* = 0.034).

### 2.4. yH2AX Was Persistently Activated 24 h after Genotoxic Exposure in ZnD Lymphocytes and ZnS AML Cells

**Figure 4:**
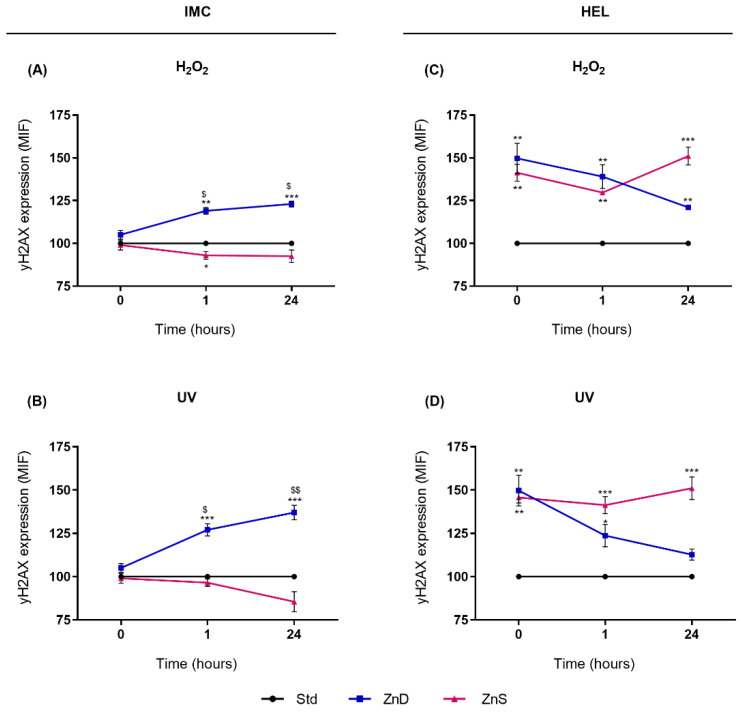
The effects of Zn depletion and supplementation on repair kinetics following genotoxic exposure in IMC (**A**,**B**) and HEL cells (**C**,**D**). Results are expressed as means ± SEM of the mean fluorescence intensity (MIF) from 4 independent experiments. MFI of ZnD and ZnS cells were normalized to Std. Note: * refers to comparison to Std and $ to 0 h, with * and $ *p* < 0.05, ** and $$ *p* < 0.01, and *** *p* < 0.001. Std, standard; ZnD, Zn-depleted; ZnS, Zn-supplemented.

To monitor repair kinetics, yH2AX expression was analyzed at several time points after genotoxic exposure in cells cultured for 15 days in Std, ZnD, and ZnS conditions. Levels of yH2AX at 1 h and 24 h after genotoxic exposure were higher in normal lymphocytes cultured in Zn depletion than in cells from Std conditions (Figure 4A,B). Moreover, in ZnD lymphocytes, yH2AX expression increased by 1.2-fold (*p* = 0.026) and 1.3-fold (*p* = 0.005) 24 h after H_2_O_2_ and UV exposure, respectively, in comparison with yH2AX expression observed right after genotoxic exposure (0 h). In ZnS lymphocytes, yH2AX expression did not significantly differ from the levels displayed by Std cells. The HEL cells presented a different kinetic repair profile (Figure 4C,D). HEL cells cultured in ZnD and ZnS conditions displayed higher yH2AX levels after genotoxic exposure than cells cultured in the Std condition. However, while a tendential decrease was observed in ZnD cells through time post-exposure, a tendential increase seemed to occur in the supplemented cells. Accordingly, 24 h after H_2_O_2_ treatment or UV radiation, expression of yH2AX was 1.5-fold higher (*p* < 0.001) in ZnS HEL cells compared to Std cells.

### 2.5. Zn Supplementation Increased Apoptosis and Decreased NDI after Genotoxic Exposure in Both Cell Lines

Cell death following genotoxic exposure increased in IMC and HEL cells supplemented with Zn comparatively to those cultured in Std Zn conditions (Supplementary Materials Figure S1), mainly due to increased apoptosis (Table 2). Specifically, the percentages of IMC cells presenting apoptotic features following H_2_O_2_ and UV exposure were 1.4-fold (*p* = 0.021) and 2.0-fold higher (*p* = 0.002) in cells supplemented with Zn for 15 days. Similarly, in HEL cells, after 15 days of Zn supplementation, exposure to H_2_O_2_ and UV radiation resulted in 2.9-fold and 3.5-fold increases (*p* = 0.033 and *p* = 0.050) in apoptotic cells.

Moreover, NDI values following genotoxic exposure were also influenced by the Zn content (Table 3). ZnS lymphocytes (IMC) displayed lower NDI values following H_2_O_2_ and UV exposure than cells from the Std condition, whereas in the leukemic cell line (HEL cells), both Zn deprivation and supplementation were related to decreased NDI values after genotoxic exposure.

### 2.6. Expression of Key DDR Genes Was Not Affected by Zn Depletion or Supplementation

Lastly, we analyzed the expression of key DDR genes in Std, ZnD, and ZnS cells after 15 days of incubation, but no significant variations were found in gene expression of IMC or HEL cells depending on the Zn content, neither in basal conditions nor post-genotoxic exposure (Supplementary Materials Tables S2–S5).

## 3. Discussion

The importance of Zn for human health is reflected by the variety of functions in which it is implicated and the large spectrum of conditions resulting from Zn deficiency [9,14]. The role of Zn in cancer is gathering more attention. Studies have revealed that Zn status is significantly compromised in cancer patients compared to healthy individuals [6,9,14,15]. In AML and other malignancies, decreases in serological or cellular Zn have been proposed as an adaptive mechanism of cells to avoid the cytoprotective effects of Zn and to acquire the biological advantages that allow malignant transformation [9,14,15,16]. This is based on the hypothesis that normal Zn levels are cytotoxic to cancer cells and that Zn deficiency allows tumor development by promoting damage tolerance, mutations, and cell survival [16]. The exact mechanisms through which Zn deficiency promotes carcinogenesis are not clear, but compelling evidence suggests that increasing dietary or supplementary Zn not only supports cancer prevention but can also limit established malignancies [6,9]. Considering the growing knowledge on DNA repair defects in leukemia, we explored the role of Zn in the modulation of the DDR in AML cells and compared responses of normal and AML cells to Zn depletion and supplementation.

Firstly, we studied the effects of Zn depletion and supplementation in the modulation of damage and cellular responses without exposure to genotoxic agents. We found that Zn deprivation increased endogenous damage, both in normal lymphocytes and AML cells, as shown by the increase in chromosomal damage biomarkers scores through time of Zn depletion. Moreover, the expression of yH2AX, a biomarker of DSB, also increased after 15 days of Zn absence in both cell lines. These findings are in line with the idea that Zn deficiency promotes genomic instability and may contribute to the acquisition of malignant phenotypes [9,17,18]. Additionally, cell viability was also influenced by Zn. While in normal lymphocytes cell death increased with Zn depletion, in AML cells, Zn supplementation rather than depletion induced a cytotoxic effect. Studies using other healthy cellular models, such as human lung fibroblasts, prostate epithelial cells, and leukocytes, previously showed that Zn deficiency promotes damage accumulation and triggers apoptosis, while supplementation reduces endogenous DNA damage and displays antiapoptotic properties [6,19,20,21,22]. In fact, the antiapoptotic effect of Zn has been recognized for a long time. In 1993, Vallee and Falchuk considered Zn as an “essentially nontoxic” metal, given its essentiality to many cellular functions and the carefully regulated mechanisms of Zn homeostasis [23]. However, the protective effects of Zn in normal cells may not manifest in the malignant cells, as shown by ours and similar studies in which Zn proved to be more cytotoxic in tumor cells than in nonmalignant [18]. Taken together, this information highlights the importance of Zn for the preservation of genome stability and cellular homeostasis and points to a cytotoxic role in malignant cells that can be explored as an anticancer therapeutic tool. Through this line of thought, Zn deficiency provides cancer cells an opportunity to thrive that would not be attainable with normal or increased Zn concentrations. However, when we evaluated the proliferation rates of AML cells, indicated by NDI calculations, these rates were significantly affected by Zn supplementation and depletion. It seems that despite Zn displaying an antiproliferative effect in AML cells, complete Zn depletion is not a condition under which cancer cells could thrive either. This result most likely reflects a limitation of Zn depletion studies, which are performed under conditions that may not exactly mimic the in vivo low-Zn conditions. While cancer cells may benefit from a Zn decrease to avoid cytotoxicity and tumor suppression, a dependence on Zn must be maintained to assure basic cellular functions.

Next, we investigated the influence of Zn in the modulation of the DDR when cells were exposed to exogenous genotoxic agents. In normal lymphocytes, Zn depletion increased chromosomal damage and compromised kinetics repair, but cell death and proliferation rates did not vary significantly, implying that an effective response to DNA damage was not achieved. Contrastingly, in supplemented lymphocytes, along with lower damage scores and a lack of noticeable changes in yH2AX expression, NDI decreased and the frequency of apoptotic cells increased. Such results may indicate an orchestrated response to genotoxic damage, including transient cell cycle arrest to provide time for damage repair and apoptosis induction to eliminate highly damaged cells. Opposite effects were found in AML cells. In these neoplastic cells, Zn supplementation increased chromosomal damage scores after genotoxic exposure, suggesting that Zn potentiates the genotoxic effect of DNA-damaging agents in malignant cells. Kinetics repair also differed substantially in supplemented AML cells compared to normal lymphocytes, since a tendential increase in yH2AX and a persistent activation 24 h post-exposure were observed. An increase in apoptosis and a decrease in NDI were also detected. From this, we postulate a dual role of Zn in the modulation of DNA damage in a cell-context-dependent manner. While in normal cells, Zn supplementation protects cells from damage accumulation and improves the DDR, in AML cells, Zn potentiates the genotoxicity of DNA-damaging agents while promoting a cytotoxic and antiproliferative effect. These conclusions are corroborated by the previously reported by Wysokinski et al. (2012), in which Zn increased the genotoxicity of anthracyclines in acute lymphoblastic leukemia cells and prevented the damaging effects in normal lymphocytes [24]. Sliwinski et al. (2009) also found a protective effect of ZnSO4 against H_2_O_2_-induced damage in normal lymphocytes and increased genotoxicity in chronic myelogenous leukemia cells [25]. In this context, the association of Zn with chemotherapy may have a dual role: increasing the cytotoxicity of therapeutic regimes in AML cells and protecting normal cells from the deleterious effects of chemotherapy drugs. However, this hypothesis needs to be tested.

To understand whether the modulatory effects of Zn were due to changes in gene expression, we studied several genes encoding sensors, mediators, and effector proteins of the DDR. We did not find a correlation between Zn levels and the expression of relevant DDR genes. Nevertheless, since Zn is required for structural, regulatory, and catalytic functions of several DDR proteins, it is reasonable that more than altering gene expression, changes in Zn bioavailability rather impact the expression and functionality of the corresponding proteins. Moreover, it should be noted that while our study had a particular interest in the modulation of DNA repair genes, other roles are ascribed to Zn that could be responsible for the reported results, which were not addressed.

In summary, and despite the mechanisms needing further characterization, our data provide evidence of the influence of Zn in the DDR either from a preventive or therapeutic perspective. Zn deprivation seems to increase the risk of spontaneous or exogenously induced DNA lesions and weaken global responses to genome damage, whereas adequate Zn levels display preventive effects. For AML management, Zn’s genotoxic, cytotoxic, and tumor suppressor roles may offer useful tools. Most treatment options in AML are based on conventional genotoxic regimens that target cancer cells by causing DNA damage [11,26,27]. However, cells may develop increased tolerance to DNA-damaging agents, ultimately leading to chemoresistance and therapy failure [26]. The reported effects may provide opportunities for Zn supplementation as part of AML treatment, with the premise that Zn may improve therapeutic outcomes by restoring efficient responses to genotoxic treatment. Moreover, its dual role in DNA damage modulation suggests that Zn may also reduce therapy-related side effects by improving the response of normal cells to the insults of genotoxic therapy while enhancing its effects in malignant cells.

## 4. Materials and Methods

### 4.1. Cell Culture

The AML cell line, HEL (human erythroleukemia), was purchased from American Type Culture Collection, and the cell line obtained from the immortalized normal human lymphocytes, IMC, was provided by the Cytogenetics and Genomics Laboratory, Faculty of Medicine, University of Coimbra. Cells were grown at initial densities of 0.4 (HEL) or 0.5 × 10^6^ cells/mL (IMC) and maintained at 37 °C in a humidified atmosphere containing 5% CO_2_. These cell lines were routinely cultured in Roswell Park Memorial Institute 1640 medium (RPMI-1640; Gibco, Invitrogen, Waltham, MA, USA), supplemented with 10% (HEL) or 20% (IMC) of heat-inactivated fetal bovine serum (FBS; Gibco, Invitrogen, Waltham, MA, USA), 2 mM of L-glutamine, 100 U/mL of penicillin, and 100 μg/mL of streptomycin (Gibco; Invitrogen). For this work, cells were cultured as mentioned above, designated as standard (Std) conditions, as well as in Zn-depleted (ZnD) and Zn-supplemented (ZnS)—40 μM of ZnSO_4_ • 7H_2_O (Sigma-Aldrich, St. Louis, MA, USA)—conditions. Zinc depletion from FBS was obtained through ion exchange resin Chelex^®^ 100 (Sigma-Aldrich, St. Louis, MA, USA) according to the manual’s instructions. ZnSO4 • 7H_2_O was resuspended in sterile water and stored at 4 °C.

### 4.2. Genotoxic Exposure

After 2, 7, and 15 days of incubation, cells were exposed to genotoxic agents, H_2_O_2_ (Sigma-Aldrich, St. Louis, MA, USA) and UV radiation, in particular to 10 µM of H_2_O_2_ for 30 min in a humidified atmosphere containing 5% of CO_2_, or to radiation for 1 min with a 6 W UV lamp at 4 mm from the light source and wavelength emission at 365 nm (Ee = 2.98 W/cm^−2^).

### 4.3. Cytokinesis-Block Micronucleus Cytome Assay

Chromosomal damage biomarkers, cell death, and nuclear division index (NDI) were evaluated by the cytokinesis-block micronucleus assay. Cells were incubated for 24 h with 4.5 µg/mL of cytochalasin-B (Sigma-Aldrich, St. Louis, MA, USA), washed twice with filtered PBS 1x, resuspended in filtered FBS, and transferred to microscopy slides. Duplicates of each condition were made, stained with Giemsa, and analyzed by light microscopy in a Nikon Eclipse 80i microscope equipped with a Nikon Digital Camera DXM 1200F (Nikon Instruments Inc., Amsterdam, Netherlands). To assess chromosomal damage, 200 viable binucleated cells were scored for the presence of micronuclei (MNi), nuclear buds (NBUDs), and nucleoplasmic bridges (NPBs). Additionally, to evaluate cytotoxicity and cytostasis, the numbers of apoptotic, necrotic, viable mononucleated, binucleated and multinucleated cells were scored from a total of 200 cells. Each endpoint was scored following the morphological criteria recommended by Fenech [28].

### 4.4. yH2AX Expression Analysis

The yH2AX expression was monitored through flow cytometry after 15 days of culture in Std, ZnD, and ZnS conditions at 0 h, 1 h, and 24 h after genotoxic exposure. Briefly, 0.5 × 10^6^ cells were washed in PBS 1x, fixed and permeabilized (Fixation and Permeabilization Kit; ImmunoStep, Salamanca, Spain), and stained with anti-yH2AX Ser139 labeled with allophycocyanin (BioLegend, San Diego, CA, USA) for 20 min at room temperature in the dark. Cells were then washed and resuspended in PBS 1x and analyzed in a FACS Calibur flow cytometer (Becton Dickinson, Franklin Lakes, NJ, USA). Here, 50,000 cells were acquired through CellQuest software (Becton Dickinson, Franklin Lakes, NJ, USA) and data were analyzed using FlowJo software (Becton Dickinson, Franklin Lakes, NJ, USA). All adequate controls to exclude autofluorescence and unspecific staining were performed.

### 4.5. Gene Expression Analysis

Total RNA was extracted from 2.5 × 10^6^ cells using a TripleXtractor (GRiSP, Porto, Portugal) and converted to cDNA with the Xpert cDNA Synthesis Mastermix (GRiSP), according to the manufacturer’s protocol. Expression of key DDR genes (*PARP1*, *XRCC1*, *OGG1*, *MSH2*, *MSH6*, *MLH1*, *XPA*, *ERCC1*, *RAD23B*, *RAD51*, *PRKDC*, *XRCC6*, *PALB2*, *FANCD2*, *MGMT*, and *TP53*) was evaluated by qPCR (Xpert Fast SYBR, GRiSP) in a QuantStudio^TM^ 3 System (ThermoFisher Scientific, Waltham, MA, USA) after 15 days of culture in each condition, before and after genotoxic exposure. The hypoxanthine phosphoribosyltransferase (*HPRT*) gene was used as the endogenous control gene. Relative gene expression was calculated using the Plaffl Method. Primer sequences are described in Supplementary Table S1.

### 4.6. Statistical Analysis

Statistical analysis was performed in GraphPad Prism 7 software (version 7.04 for Windows; GraphPad Software, Inc. San Diego, CA, USA). Differences between conditions were determined using ordinary one-way ANOVA followed by Dunnett’s multiple comparisons test or Kruskal–Wallis test followed by Dunn’s multiple comparisons test. Changes through time were assessed by one-way ANOVA for repeated measures followed by Dunnett’s multiple comparisons test or Friedman’s test followed by Dunn’s multiple comparisons test. A significance level of *p* < 0.05 was considered statistically significant. Data are expressed as means ± SEM of the number of independent experiments performed.

## Figures and Tables

**Table 1:** Modulation of nuclear division indexes of normal lymphocytes and AML cells unexposed to genotoxic agents by Zn depletion and supplementation.

	IMC			HEL		
	2nd Day	7th Day	15th Day	2nd Day	7th Day	15th Day
Std	1.61 ± 0.05	1.46 ± 0.03	1.51 ± 0.02	1.79 ± 0.05	1.76 ± 0.02	1.67 ± 0.03
ZnD	1.49 ± 0.03	1.35 ± 0.01	1.41 ± 0.04	1.73 ± 0.01	1.57 ± 0.07 *	1.53 ± 0.03 *
ZnS	1.61 ± 0.02	1.57 ± 0.02	1.54 ± 0.03	1.69 ± 0.02 *	1.61 ± 0.04 *	1.49 ± 0.03 **^,$^

Results are expressed as means ± SEM of NDI from 6 independent experiments. Note: * refers to comparison to Std and ^$^ to 2nd day, with * and ^$^ *p* < 0.05 and ** *p* < 0.01. Std, standard; ZnD, Zn-depleted; ZnS, Zn-supplemented.

**Table 2:** Modulation of apoptosis by Zn depletion and supplementation following genotoxic exposure.

	IMC			HEL		
	2nd Day	7th Day	15th Day	2nd Day	7th Day	15th Day
**H_2_O_2_**						
Std	6.0 ± 0.9	7.5 ± 0.8	9.2 ± 3.8	6.8 ± 2.1	5.5 ± 0.8	4.8 ± 1.9
ZnD	4.0 ± 2.6	3.8 ± 1.5	8.2 ± 1.8	6.2 ± 0.3	8.2 ± 2.8	9.0 ± 3.8
ZnS	7.2 ± 0.7	9.8 ± 1.7	12.8 ± 3.0 *	15.3 ± 4.0	15.7 ± 0.8 *	13.8 ± 2.9 *
**UV**						
Std	8.7 ± 1.2	5.7 ± 1.0	8.8 ± 1.5	4.2 ± 1.4	4.8 ± 1.8	5.2 ± 0.7
ZnD	5.5 ± 0.8	5.5 ± 0.5	6.0 ± 1.7	6.5 ± 1.2	5.7 ± 1.5	10.7 ± 6.0
ZnS	12.5 ± 1.2	14.8 ± 2.4	17.5 ± 1.7 ***	7.2 ± 3.9	12.0 ± 2.1 *	18.2 ± 2.2 *

Results are expressed as means ± SEM of percentages of apoptotic cells from 6 independent experiments. Note: * refers to comparison to Std, with * *p* < 0.05, and *** *p* < 0.001. H_2_O_2_, hydrogen peroxide; UV, ultraviolet radiation; Std, standard; ZnD, Zn-depleted; ZnS, Zn-supplemented.

**Table 3:** Modulation of nuclear division indexes of normal lymphocytes and AML cells exposed to genotoxic agents by Zn depletion and supplementation.

	IMC			HEL		
	2nd Day	7th Day	15th Day	2nd Day	7th Day	15th Day
**H_2_O_2_**						
Std	1.40 ± 0.02	1.35 ± 0.01	1.24 ± 0.03	1.63 ± 0.05	1.68 ± 0.01	1.62 ± 0.04
ZnD	14.1 ± 0.04	1.38 ± 0.03	1.26 ± 0.02	1.46 ± 0.04	1.33 ± 0.05 *	1.35 ± 0.03 ***
ZnS	1.37 ± 0.02	1.37 ± 0.02	1.35 ± 0.03	1.43 ± 0.02	1.54 ± 0.07 *	1.46 ± 0.04
**UV**						
Std	1.45 ± 0.02	1.33 ± 0.01	1.34 ± 0.02	1.71 ± 0.03	1.59 ± 0.02	1.52 ± 0.01
ZnD	1.40 ± 0.02	1.30 ± 0.02	1.36 ± 0.03	1.66 ± 0.07	1.28 ± 0.03 *^$^	1.13 ± 0.02 **^$^
ZnS	1.22 ± 0.01 ***	1.21 ± 0.04	1.16 ± 0.02 *	1.64 ± 0.02 *	1.58 ± 0.02	1.37 ± 0.04

Results are expressed as means ± SEM of NDI from 6 independent experiments. Note: * refers to comparison to Std and ^$^ to 2nd day, with * and ^$^ *p* < 0.05, ** *p* < 0.01, and *** *p* < 0.001. Std, standard; ZnD, Zn-depleted; ZnS, Zn-supplemented.

## Data Availability

All data generated or analyzed during this study are included in this published article.

## Supplementary Materials

The following supporting information can be downloaded at: https://www.mdpi.com/article/10.3390/ijms23052567/s1.

## References

[1] Ciccia A., Elledge S.J. (2010). The DNA Damage Response: Making It Safe to Play with Knives. Mol. Cell.

[2] Fenech M.F. (2010). Dietary reference values of individual micronutrients and nutriomes for genome damage prevention: Current status and a road map to the future. Am. J. Clin. Nutr..

[3] Collins A.R., Azqueta A., Langie S. (2012). Effects of micronutrients on DNA repair. Eur. J. Nutr..

[4] Reddy V.S., Palika R., Ismail A., Pullakhandam R., Reddy G.B. (2018). Nutrigenomics: Opportunities & challenges for public health nutrition. Indian J. Med. Res..

[5] Tehrani S.S., Hosseini H.M., Yousefi T., Abolghasemi M., Qujeq D., Maniati M., Amani J. (2018). The crosstalk between trace elements with DNA damage response, repair, and oxidative stress in cancer. J. Cell. Biochem..

[6] Dhawan D.K., Chadha V.D. (2010). Zinc: A promising agent in dietary chemoprevention of cancer. Indian J. Med. Res..

[7] Orlov A.P., Orlova M.A., Trofimova T.P., Kalmykov S.N., Kuznetsov D.A. (2018). The role of zinc and its compounds in leu-kemia, Journal of biological inorganic chemistry. J. Biol. Inorg. Chem..

[8] Skrajnowska D., Bobrowska-Korczak B. (2019). Role of Zinc in Immune System and Anti-Cancer Defense Mechanisms. Nutrients.

[9] Ho E. (2004). Zinc deficiency, DNA damage and cancer risk. J. Nutr. Biochem..

[10] Delia D., Mizutani S. (2017). The DNA damage response pathway in normal hematopoiesis and malignancies. Int. J. Hematol..

[11] Nilles N., Fahrenkrog B. (2017). Taking a Bad Turn: Compromised DNA Damage Response in Leukemia. Cells.

[12] Valadbeigi S., Javadian S., Ebrahimi-Rad M., Khatami S., Saghiri R. (2019). Assessment of trace elements in serum of acute lymphoblastic and myeloid leukemia patients. Exp. Oncol..

[13] Zhu B., Wang J.Y., Zhou J.J., Zhou F., Cheng W., Liu Y.T., Wang J., Chen X., Chen D.H., Luo L. (2017). PML-RARalpha stabilized by zinc in human acute promyelocytic leukemia NB4 cells. J. Inorg. Biochem..

[14] Huang L., Drake V.J., Ho E. (2015). Zinc. Adv. Nutr..

[15] Hoang B.X., Han B., Shaw D.G., Nimni M.E. (2016). Zinc as a possible preventive and therapeutic agent in pancreatic, prostate, and breast cancer. Eur. J. Cancer Prev..

[16] Raisi A., Mehrzad V., Mahmood-Zadeh M., Feizi A. (2018). Determination Relation of the Zinc Serum Level in Acute Leukemia Adult Patients with Mucositis and Neutropenic Prevalence before and after Treatment in Isfahan’ Seyed-Al-Shohada Hospital, 2012–2013. Adv. Biomed. Res..

[17] Yildiz A., Kaya Y., Tanriverdi O. (2019). Effect of the Interaction Between Selenium and Zinc on DNA Repair in Association With Cancer Prevention. J. Cancer Prev..

[18] Franklin R.B., Costello L.C. (2009). The important role of the apoptotic effects of zinc in the development of cancers. J. Cell. Biochem..

[19] Ho E., Courtemanche C., Ames B.N. (2003). Zinc Deficiency Induces Oxidative DNA Damage and Increases P53 Expression in Human Lung Fibroblasts. J. Nutr..

[20] Yan M., Song Y., Wong C.P., Hardin K., Ho E. (2008). Zinc Deficiency Alters DNA Damage Response Genes in Normal Human Prostate Epithelial Cells. J. Nutr..

[21] Reaves S.K., Fanzo J., Arima K., Wu J.Y.J., Wang Y.R., Lei K.Y. (2000). Expression of the p53 tumor suppressor gene is up-regulated by depletion of intracellular zinc in HepG2 cells. J. Nutr..

[22] Zyba S.J., Shenvi S.V., Killilea D.W., Holland T.C., Kim E., Moy A., Sutherland B., Gildengorin V., Shigenaga M.K., King J.C. (2017). A moderate increase in dietary zinc reduces DNA strand breaks in leukocytes and alters plasma proteins without changing plasma zinc concentrations. Am. J. Clin. Nutr..

[23] Vallee B.L., Falchuk K.H. (1993). The biochemical basis of zinc physiology. Physiol. Rev..

[24] Wysokinski D., Blasiak J., Woźniak K. (2012). Zinc differentially modulates DNA damage induced by anthracyclines in normal and cancer cells. Exp. Oncol..

[25] Śliwiński T., Czechowska A., Kolodziejczak M., Jajte J., Wiśniewska-Jarosińska M., Blasiak J. (2009). Zinc salts differentially modulate DNA damage in normal and cancer cells. Cell Biol. Int..

[26] D’Andrea A.D. (2010). Targeting DNA repair pathways in AML. Best Pr. Res. Clin. Haematol..

[27] Villela L., Bolanos-Meade J. (2011). Acute myeloid leukaemia: Optimal management and recent developments. Drugs.

[28] Fenech M. (2007). Cytokinesis-block micronucleus cytome assay. Nat. Protoc..

