# Supplementary material for: Zinc Prevents DNA Damage in Normal Cells but Shows Genotoxic and Cytotoxic Effects in Acute Myeloid Leukemia Cells
Source: Int J Mol Sci. 2022 Feb 25;23(5):2567. doi: 10.3390/ijms23052567 (PMC8910549; doi:10.3390/ijms23052567)
Supplement: Supplementary file 1 [file ijms-23-02567-s001.zip › ijms-1594823-supplementary.pdf]

SUPPLEMENTARY MATERIAL

Supplementary Table S1. Target genes, primer sequences, and qPCR conditions.

Gene	Primer sequence	[Primers]	$T_{annealing}$
PARP1	Forward: CGGAGTCTTCGGATAAGCTCT<br>Reverse: TTTCCATCAAACATGGGCGAC	200nM	60ºC
XRCC1	Forward: TCAAGGCAGACACTTACCGAA<br>Reverse: TCCAACTGTAGGACCACAGAG	150nM	60ºC
OGG1	Forward: ATCGTACTCTAGCCTCCACT<br>Reverse: TGAGCCAGGGTAACATCTAG	75nM	61ºC
MSH2	Forward: AGTCAGAGCCCTTAACCTTTTTC<br>Reverse: GAGAGGCTGCTTAATCCACTG	100nM	60ºC
MSH6	Forward: CCAAGGCGAAGAACCTCAAC<br>Reverse: ACCAGGGGTAACCCTCCATC	150nM	60ºC
MLH1	Forward: CTCTTCATCAACCATCGTCTGG<br>Reverse: GCAAATAGGCTGCATACACTGTT	150nM	60ºC
XPA	Forward: CCAGGACCTGTTATGGAATTTGA<br>Reverse: GCTTCTTGACTACCCCAAACTTC	75nM	61ºC
ERCC1	Forward: CTACGCCGAATATGCCATCTC<br>Reverse: GTACGGGATTGCCCCTCTG	100nM	60ºC
RAD23B	Forward: AGCAACTGACAGTACATCGGG<br>Reverse: CTCGTTCATAGCCCATTGACAT	50nM	60ºC
RAD51	Forward: CAACCCATTTCACGGTTAGAGC<br>Reverse: TTCTTTGGCGCATAGGCAACA	150nM	60ºC
PRKDC	Forward: CTGTGCAACTTCACTAAGTCCA<br>Reverse: CAATCTGAGGACGAATTGCCT	150nM	60ºC
XRCC6	Forward: GTTGATGCCTCCAAGGCTATG<br>Reverse: CCCCTTAAACTGGTCAAGCTCTA	150nM	60ºC
PALB2	Forward: ATCCTGCTAGATCACCAGTAACT<br>Reverse: TCAAGGTGCTGACTACTACCG	75nM	60ºC
FANCD2	Forward: ACATACCTCGACTCATTGTCAGT<br>Reverse: TCGGAGGCTTGAAAGGACATC	150nM	60ºC
MGMT	Forward: TTTTCCAGCAAGAGTCGTTCAC<br>Reverse: GGGACAGGATTGCCTCTCAT	150nM	60ºC
TP53	Forward: CAGCACATGACGGAGGTTGT<br>Reverse: TCATCCAAATACTCCACACGC	75nM	60ºC

[Primers] – Primers concentration; $T_{annealing}$ – annealing temperature.

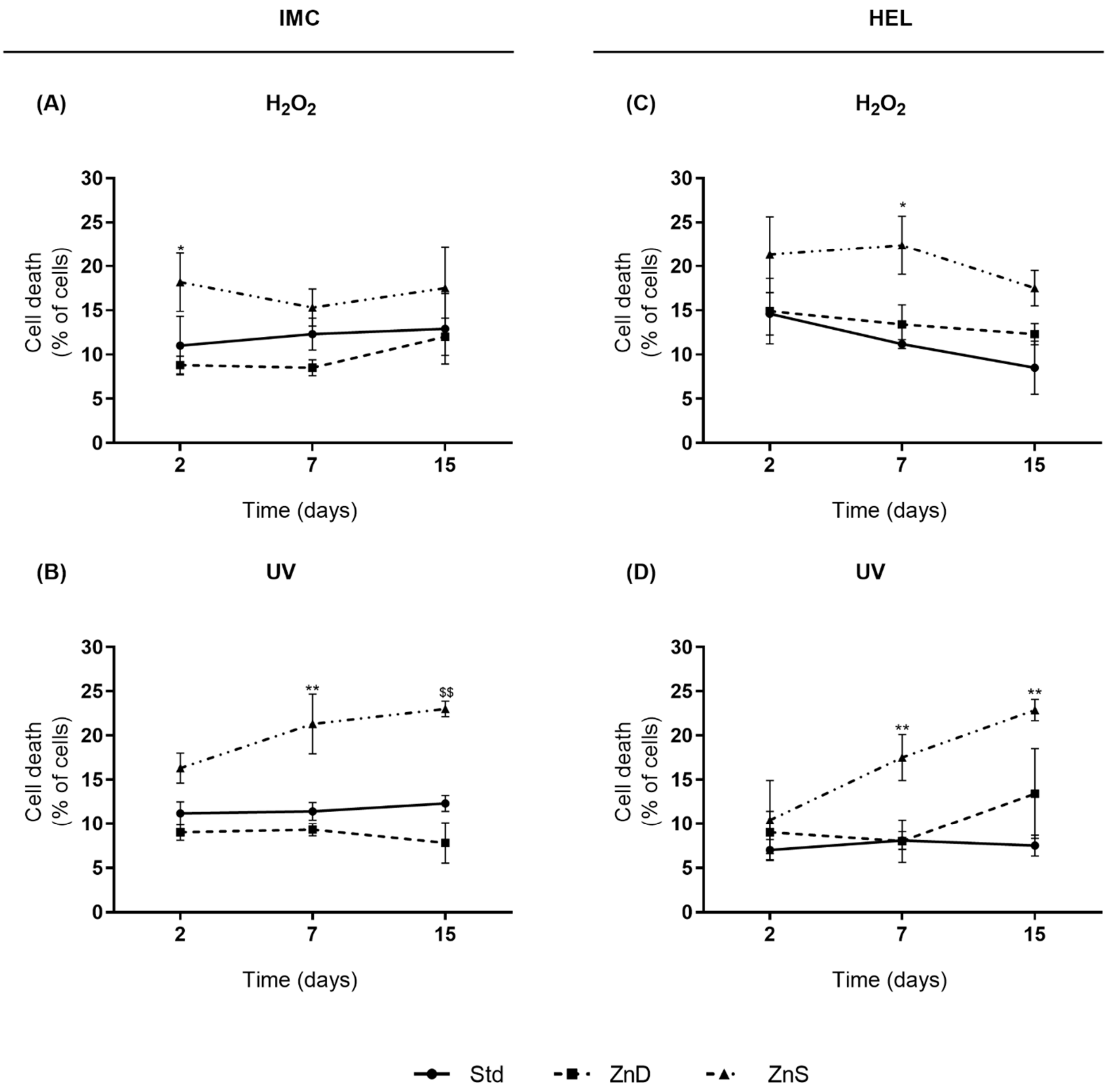

Supplementary Figure S1. The effects of Zn depletion and supplementation in the modulation of chromosomal damage following genotoxic exposure in IMC (A, B) and HEL cells (C, D). Results are expressed as mean ± SEM of the percentage of cells displaying morphological features of apoptosis and necrosis from 6 independent experiments. * refers to comparison to Std and $^{\$}$ to 2nd day, with * $p<0.05$, and ** and $^{\$\$}$ $p<0.01$. Std, Standard; ZnD, Zn-depleted; ZnS, Zn-supplemented.

Supplementary Table S2. Expression of DDR genes in IMC cells unexposed to genotoxic agents.

	Std	ZnD	ZnS
PARP1	0.97 ± 0.02	0.95 ± 0.02	0.95 ± 0.02
XRCC1	0.95 ± 0.02	0.92 ± 0.02	0.92 ± 0.02
OGG1	0.88 ± 0.01	0.83 ± 0.02	0.87 ± 0.02
MSH2	0.96 ± 0.03	0.92 ± 0.03	0.92 ± 0.03
MSH6	0.85 ± 0.01	0.87 ± 0.02	0.85 ± 0.01
MLH1	0.98 ± 0.01	0.98 ± 0.01	0.98 ± 0.01
XPA	0.95 ± 0.02	0.93 ± 0.02	0.93 ± 0.02
ERCC1	0.76 ± 0.01	0.72 ± 0.01	0.74 ± 0.01
RAD23B	0.90 ± 0.04	0.84 ± 0.04	0.83 ± 0.05
RAD51	0.90 ± 0.03	0.91 ± 0.03	0.93 ± 0.03
PRKDC	0.87 ± 0.02	0.89 ± 0.02	0.88 ± 0.02
XRCC6	0.99 ± 0.01	0.98 ± 0.01	1.00 ± 0.01
PALB2	0.80 ± 0.02	0.78 ± 0.02	0.77 ± 0.03
FANCD2	0.86 ± 0.01	0.86 ± 0.02	0.85 ± 0.01
MGMT	0.88 ± 0.02	0.88 ± 0.02	0.86 ± 0.02
TP53	0.94 ± 0.02	0.93 ± 0.01	0.91 ± 0.02

IMC cells are incubated during 15 days in 3 conditions, Std, ZnD and ZnS. Results are expressed as relative gene expression normalized to *HPRT*, calculated through the Plaffl Method. Data are expressed as mean ± SEM of 5 independent experiments. H_2O_2, hydrogen peroxide; UV, ultraviolet radiation; Std, Standard; ZnD, Zn-depleted; ZnS, Zn-supplemented.

Supplementary Table S3. Expression of DDR genes in HEL cells unexposed to genotoxic agents.

	Std	ZnD	ZnS
PARP1	0.99 ± 0.01	1.00 ± 0.01	0.99 ± 0.01
XRCC1	0.95 ± 0.01	0.95 ± 0.01	0.95 ± 0.01
OGG1	0.82 ± 0.02	0.86 ± 0.01	0.80 ± 0.02
MSH2	0.97 ± 0.01	0.97 ± 0.01	0.97 ± 0.01
MSH6	0.83 ± 0.03	0.83 ± 0.03	0.82 ± 0.04
MLH1	0.93 ± 0.01	0.92 ± 0.01	0.93 ± 0.01
XPA	0.95 ± 0.02	0.96 ± 0.01	0.96 ± 0.01
ERCC1	0.91 ± 0.04	0.93 ± 0.03	0.93 ± 0.03
RAD23B	0.84 ± 0.07	0.84 ± 0.07	0.85 ± 0.07
RAD51	0.92 ± 0.01	0.92 ± 0.02	0.93 ± 0.01
PRKDC	0.96 ± 0.01	0.96 ± 0.01	0.95 ± 0.01
XRCC6	1.04 ± 0.01	1.02 ± 0.01	1.01 ± 0.01
PALB2	0.83 ± 0.05	0.83 ± 0.05	0.83 ± 0.05
FANCD2	0.97 ± 0.01	0.97 ± 0.01	0.97 ± 0.01
MGMT	0.95 ± 0.01	0.95 ± 0.01	0.95 ± 0.01
TP53	0.99 ± 0.02	1.00 ± 0.01	0.99 ± 0.01

HEL cells are incubated during 15 days in 3 conditions, Std, ZnD and ZnS. Results are expressed as relative gene expression normalized to *HPRT*, calculated through the Plaffl Method. Data are expressed as mean ± SEM of 5 independent experiments. H_2O_2, hydrogen peroxide; UV, ultraviolet radiation; Std, Standard; ZnD, Zn-depleted; ZnS, Zn-supplemented.

Supplementary Table S4. Expression of DDR genes following genotoxic exposure in IMC cells.

	H_2O_2			UV		
	**Std**	**ZnD**	**ZnS**	**Std**	**ZnD**	**ZnS**
PARP1	0.95 ± 0.02	0.94 ± 0.02	0.96 ± 0.02	0.95 ± 0.02	0.95 ± 0.01	0.95 ± 0.03
XRCC1	0.91 ± 0.02	0.91 ± 0.02	0.92 ± 0.02	0.92 ± 0.03	0.91 ± 0.02	0.91 ± 0.02
OGG1	0.85 ± 0.02	0.84 ± 0.01	0.84 ± 0.02	0.87 ± 0.02	0.87 ± 0.04	0.85 ± 0.03
MSH2	0.90 ± 0.03	0.91 ± 0.03	0.90 ± 0.03	0.92 ± 0.03	0.91 ± 0.03	0.90 ± 0.03
MSH6	0.82 ± 0.01	0.85 ± 0.04	0.82 ± 0.01	0.82 ± 0.01	0.82 ± 0.01	0.82 ± 0.01
MLH1	0.96 ± 0.02	0.97 ± 0.01	0.96 ± 0.01	0.96 ± 0.02	0.97 ± 0.01	0.96 ± 0.02
XPA	0.92 ± 0.02	0.92 ± 0.03	0.92 ± 0.02	0.89 ± 0.05	0.91 ± 0.02	0.92 ± 0.02
ERCC1	0.75 ± 0.02	0.73 ± 0.02	0.76 ± 0.02	0.75 ± 0.02	0.76 ± 0.02	0.77 ± 0.02
RAD23B	0.83 ± 0.04	0.82 ± 0.05	0.84 ± 0.04	0.83 ± 0.04	0.83 ± 0.05	0.84 ± 0.04
RAD51	0.94 ± 0.03	0.92 ± 0.04	0.94 ± 0.03	0.93 ± 0.03	0.92 ± 0.04	0.94 ± 0.03
PRKDC	0.90 ± 0.01	0.89 ± 0.02	0.90 ± 0.02	0.87 ± 0.03	0.90 ± 0.02	0.90 ± 0.02
XRCC6	0.97 ± 0.02	1.00 ± 0.01	0.98 ± 0.02	0.95 ± 0.03	0.99 ± 0.02	0.98 ± 0.02
PALB2	0.77 ± 0.02	0.77 ± 0.01	0.76 ± 0.01	0.77 ± 0.01	0.78 ± 0.02	0.76 ± 0.01
FANCD2	0.85 ± 0.01	0.85 ± 0.01	0.86 ± 0.01	0.85 ± 0.01	0.84 ± 0.01	0.84 ± 0.01
MGMT	0.83 ± 0.03	0.81 ± 0.02	0.84 ± 0.03	0.84 ± 0.03	0.83 ± 0.01	0.84 ± 0.03
TP53	0.93 ± 0.01	0.91 ± 0.01	0.92 ± 0.01	0.94 ± 0.01	0.92 ± 0.02	0.96 ± 0.01

IMC cells are incubated during 15 days in 3 conditions, Std, ZnD and ZnS. Results are expressed as relative gene expression normalized to *HPRT*, calculated through the Plaffl Method. Data are expressed as mean ± SEM of 5 independent experiments. H_2O_2, hydrogen peroxide; UV, ultraviolet radiation; Std, Standard; ZnD, Zn-depleted; ZnS, Zn-supplemented.

Supplementary Table S5. Expression of DDR genes following genotoxic exposure in HEL cells.

	H_2O_2			UV		
	Std	ZnD	ZnS	Std	ZnD	ZnS
PARP1	1.00 ± 0.01	1.01 ± 0.02	0.99 ± 0.01	1.01 ± 0.01	1.02 ± 0.01	1.01 ± 0.01
XRCC1	0.96 ± 0.01	0.95 ± 0.01	0.95 ± 0.01	0.95 ± 0.01	0.94 ± 0.01	0.98 ± 0.03
OGG1	0.84 ± 0.01	0.83 ± 0.01	0.80 ± 0.02	0.78 ± 0.02	0.82 ± 0.01	0.81 ± 0.01
MSH2	0.97 ± 0.01	0.98 ± 0.01	0.97 ± 0.01	0.97 ± 0.01	0.97 ± 0.01	0.97 ± 0.01
MSH6	0.83 ± 0.03	0.85 ± 0.03	0.83 ± 0.03	0.82 ± 0.03	0.81 ± 0.02	0.83 ± 0.03
MLH1	0.93 ± 0.01	0.93 ± 0.01	0.95 ± 0.01	0.92 ± 0.01	0.92 ± 0.01	0.92 ± 0.01
XPA	0.96 ± 0.01	0.96 ± 0.01	0.96 ± 0.01	0.95 ± 0.01	0.95 ± 0.01	0.96 ± 0.01
ERCC1	0.94 ± 0.02	0.94 ± 0.02	0.94 ± 0.02	0.92 ± 0.02	0.92 ± 0.02	0.91 ± 0.02
RAD23B	0.86 ± 0.04	0.86 ± 0.05	0.86 ± 0.06	0.85 ± 0.05	0.85 ± 0.04	0.85 ± 0.06
RAD51	0.94 ± 0.01	0.92 ± 0.01	0.94 ± 0.01	0.94 ± 0.01	0.93 ± 0.01	0.94 ± 0.01
PRKDC	0.96 ± 0.01	0.96 ± 0.01	0.95 ± 0.01	0.94 ± 0.01	0.96 ± 0.01	0.94 ± 0.02
XRCC6	1.01 ± 0.03	1.01 ± 0.03	1.03 ± 0.02	0.98 ± 0.02	0.97 ± 0.03	1.00 ± 0.02
PALB2	0.83 ± 0.03	0.83 ± 0.04	0.83 ± 0.04	0.80 ± 0.04	0.82 ± 0.03	0.82 ± 0.04
FANCD2	0.97 ± 0.01	0.96 ± 0.02	0.96 ± 0.01	0.94 ± 0.02	0.95 ± 0.02	0.96 ± 0.01
MGMT	0.95 ± 0.01	0.95 ± 0.01	0.95 ± 0.01	0.95 ± 0.01	0.95 ± 0.01	0.95 ± 0.01
TP53	0.99 ± 0.01	0.99 ± 0.01	0.98 ± 0.01	0.99 ± 0.01	0.99 ± 0.01	0.99 ± 0.01

HEL cells are incubated during 15 days in 3 conditions, Std, ZnD and ZnS. Results are expressed as relative gene expression normalized to *HPRT*, calculated through the Plaffl Method. Data are expressed as mean ± SEM of 5 independent experiments. H_2O_2, hydrogen peroxide; UV, ultraviolet radiation; Std, Standard; ZnD, Zn-depleted; ZnS, Zn-supplemented.
